# Future Trends in Worldwide River Nitrogen Transport and Related Nitrous Oxide Emissions: A Scenario Analysis

**DOI:** 10.1100/tsw.2001.279

**Published:** 2001-11-02

**Authors:** Carolien Kroeze, Sybil P. Seitzinger, Rita Domingues

**Affiliations:** Environmental Systems Analysis Group, Wageningen University, The Netherlands

**Keywords:** nitrogen, nitrous oxide, world rivers, estuaries, future trends, biogeochemical N cycling, agriculture, fertilizer

## Abstract

We analyze possible future trends in dissolved inorganic nitrogen (DIN) export by world rivers and associated emissions of nitrous oxide (N2O). Our scenarios either assume that current trends continue or that nitrogen (N) inputs to aquatic systems are reduced as a result of changes in agriculture practices and fuel combustion technologies. The results indicate that moderate changes in the human diet in North America and Europe, reducing worldwide fertilizer use by only 16%, relative to Business-as-Usual (BAU) levels, may reduce DIN export rates to the North Atlantic and European Seas by about one third and associated N2O emissions by 36 to 77%. We furthermore calculate that relatively large reductions in NOy deposition rates in Europe (of about 80%) may reduce DIN export by rivers by a moderate 8% or less, relative to BAU levels. The potential effect of reduced NOy deposition on riverine DIN export is moderate, because most N in European rivers stems from agriculture, and not from fuel combustion. Nevertheless, the calculated 9% reduction (relative to BAU) in DIN inputs to the North Sea as a potential side effect of air pollution control may help achieve the international policy targets for reduced N inputs to the North Sea.

## INTRODUCTION

Human activities have altered the global nitrogen (N) cycle[1,2]. Model calculations indicate that, worldwide, rivers transported about 20 Tg of dissolved inorganic nitrogen (DIN) to the coastal oceans in 1990[3]. Roughly 25% of this amount may be considered natural, indicating that human activities have increased DIN export rates severalfold above the natural levels. A major source of this increased N is agricultural N, entering rivers due to leaching and runoff, or through point sources. In addition, N oxides emitted during fuel combustion may end up in rivers after atmospheric deposition in the watersheds. Increased N availability increases aquatic nitrification and denitrification. As a result, emissions of the greenhouse gas nitrous oxide ($N_2O$) from rivers, estuaries, and continental shelves are presently higher than in preindustrial times. Worldwide, over 75% of anthropogenic $N_2O$ emissions may be associated with N losses from agriculture, and up to one third of this $N_2O$ may be formed in natural (mainly aquatic) systems after leaching or other losses of agricultural N from agricultural systems[4].

Analyses of expected future trends indicate that riverine DIN export and aquatic $N_2O$ emissions may increase considerably in the coming decades[5,6]. Here we present results of model runs in which alternative future developments are considered, along with their effect on DIN export by world rivers and associated $N_2O$ emissions. Our scenarios assume either that current trends continue or that N inputs to aquatic systems are reduced as a result of changes in agriculture practices and fuel combustion technologies. Global as well as regional results are presented.

## DESCRIPTION OF THE N-MODEL

We used the N-model described by Seitzinger and Kroeze[3]. The N-model calculates aquatic emissions of $N_2O$ as a function of nitrification and denitrification rates in rivers, estuaries, and continental shelves, which, in turn, are a function of human activities in watersheds. $N_2O$ emissions are nonlinearly related to DIN export rates in the model, with higher emission factors for higher N levels in aquatic systems. The N-model is a global model and includes 177 watersheds that transport their water to 303 estuarine gridcells. Calculations are either performed on a grid of 1° longitude by 1° latitude (e.g., $N_2O$ emissions), or at the watershed level (e.g., N transport by rivers). Inputs to the model include water runoff, urban population, fertilizer use, and $NO_y$ deposition in exoreic watersheds. (Watersheds draining to seas and oceans; watersheds that drain into inland drainage basins (endoreic runoff) are not included in most model calculations.) Model output includes DIN export by rivers to the world's oceans, and associated emissions of $N_2O$ from rivers, estuaries, and continental shelves. Here we focus on emissions from rivers and estuaries, on a watershed level.

The N-model calculates DIN export by rivers as a function of human activities in the watersheds. The basis of the model is a statistical analysis by Caraco and Cole[7] of the relationship between nitrate export by rivers and fertilizer use, urban population, and $NO_y$ deposition in the watersheds. The N-model does not explicitly use manure applied to soils and biological $N_2$ fixation as a model input to calculate DIN export by rivers. However, it implicitly accounts for these N inputs. The amount of fertilizer use in the watershed may be considered an indicator for total agricultural activities giving rise to N loadings to aquatic systems.

## SCENARIO DESCRIPTION

We present results for three scenarios for the year 2050 (Box 1).

### Business-as-Usual Scenario (BAU)

The BAU scenario is taken from Kroeze and Seitzinger[5] and assumes that the population, fertilizer use, and $NO_y$ deposition rates by 2050 will be higher than in 1990. Projections for the 2050 population were taken from the United Nations[8], for fertilizer use from Bouwman[9] and Kreileman and Bouwman[10], and for atmospheric deposition from Dentener (personal communication). Other N-model inputs (e.g., water runoff) and parameters were assumed to remain at their 1990 levels.

### Low N Diet Scenario (DIET)

The Low N Diet scenario explores the potential effect of a change in the human diet on DIN export by rivers and the associated $N_2O$ emissions. N requirements in agriculture are related to the human diet. The BAU 2050 projections indicate that the worldwide increase in fertilizer use between 1990 and 2050 (145%) will exceed the population growth (70%). This is because the increasing N inputs to agriculture are the result not only of a growing population but also of a change in the human diet, which is increasingly based on animal proteins. In general, animal production requires more N inputs than crop production. For Norway, it was estimated that animals require 7 kg N in feed to supply 1 kg N in wholesale edible products like beef or milk[11], while the production of animal feed requires similar amounts of N inputs as the production of arable crops. This illustrates that a shift in human consumption from plant to animal proteins increases N use in agriculture. For instance, Bleken[11] showed that the N inputs needed in agriculture may increase with increasing consumption of animal proteins. She calculated that a diet similar to that in Italy in 1963 or Turkey in 1993 would require N application to soils (from synthetic fertilizers, animal manure, atmospheric deposition, and biological $N_2$ fixation) of about 40 kg N/person/year. This is low, compared to inputs of 70 kg N/person/year in many industrialized regions today. These low N diets still include considerable meat consumption (animal protein intake of about 30 g/person/day), but less than in many industrialized countries at present (up to 70 g/person/day).

In the DIET scenario, we assume that the human diet in regions that today are industrialized will change towards a larger share of plant proteins than assumed in the BAU scenario for 2050. We assume that the 2050 synthetic fertilizer use in the DIET scenario is reduced, relative to the 2050 BAU level, as a result of these dietary changes. These levels were tentatively defined, partly based on an interpretation of the study by Bleken[11].

Bleken's estimate of N application to soils of 40 kg N/person/year includes inputs from synthetic fertilizer, animal manure, biological $N_2$ fixation, and atmospheric deposition. The required changes in agricultural practices to arrive at N inputs of 40 kg N/person/year are complex, and there may be several strategies to achieve this aim. Here, we made simplifying assumptions. Our analysis must therefore be considered a first step in analyzing the potential effects of dietary changes. For regions shifting to a lower N diet in the DIET scenario, we assumed that the reduction in N inputs to watersheds, relative to the BAU scenario, is only caused by a reduction in synthetic fertilizer use. We also assumed that, in the DIET scenario, atmospheric deposition and biological $N_2$ fixation equal their 2050 BAU levels. The dietary changes in the DIET scenario will also affect the number of livestock and, as a result, the available livestock manure. We tentatively assumed that, in regions where we assume dietary changes, the availability of organic fertilizers will remain at the 1990 level. This may, however, not be realistic for all world regions and may, in fact, imply import or export of manure.

To estimate the reductions in synthetic fertilizer associated with a limit to N inputs to soils of 40 kg N/person/year, we first calculated per capita N inputs to soils of the watersheds included in our model (Fig. 1). The N inputs are from fertilizer (BAU 2050 values), manure (assumed to stay at the 1990 level), and $NO_y$ deposition (BAU 2050 values). These N inputs exceed 40 kg N/person/year for exoreic watersheds in North and South America, Europe, and Northeast Asia. From this information, we calculated the reductions in synthetic fertilizer use, relative to 2050 BAU levels, that would be required so that N inputs to soils do not exceed 40 kg N/person/year. This resulted in assumed reductions of 80% for North America, 60% for Europe, Australia, New Zealand, the Middle East, and Japan, and 10% for Northeast Asia. Although the assumed reductions in per capita fertilizer use relative to the 2050 BAU scenario may seem high, they do not reduce synthetic fertilizer use compared to 1990 in most world regions, except in Europe (by about 45%) and in North America (by about 75%). Total calculated N inputs for Europe and North America are 22 and 34% lower, respectively, than in 1990. The difference between Europe and North America is the net effect of assumed differences in human diet, number of people, and livestock numbers. A more realistic interpretation of Bleken's low N diets for North America would probably be that livestock numbers decrease relative to 1990 levels, while fertilizer use is somewhat higher than assumed here. The net effect hereof on calculated DIN rates for the DIET scenario would, however, be small.

## Scenario for Low $NO_y$ Deposition in Europe (NDEP)

In the NDEP scenario, we analyze the effect of reductions in $NO_y$ deposition in Europe on DIN export by rivers. To this end, we first calculated regional patterns of $NO_y$ deposition using the *R*egional *A*ir pollution *IN*formation *S*ystem, RAINS[12,13]. RAINS includes projections for future fuel use by sector and region in Europe, as well as information on technologies to reduce these emissions. We used RAINS version 7.02 to calculate $NO_y$ deposition patterns for a scenario combining the RAINS *Official Energy Pathway* for future fuel use and the RAINS *maximum feasible reduction strategy* for reducing emissions of nitrogen oxides. The resulting scenario assumes that fuel use will develop following official country projections, as available in RAINS 7.02, and that, meanwhile, all countries will apply maximum emission control in electricity generation, transport, and industry. This reflects the technical potential to reduce $NO_x$ emissions in Europe. The associated costs of such reductions are relatively high[12,13]. RAINS calculates atmospheric $NO_y$ deposition patterns, taking into account the location of emissions and atmospheric transport. These $NO_y$ deposition rates were used as input to the N-model to calculate DIN export rates by rivers. The conversion of RAINS output ($NO_y$ deposition rates for EMEP grid cells of 150 by 150 km) to N-model input (average deposition rates by watershed region) was done by assigning RAINS grid cells to N-model watershed regions (see Domingues[14] for details on data conversion). Undertaking this scenario analysis was complicated by the fact that RAINS does not generate results for the year 2050, but for 2010. For the 2050 NDEP scenario, we therefore assume that $NO_y$ deposition rates remain at their reduced 2010 level, which would represent a maximum reduction. It should be noted that the uncontrolled $NO_y$ deposition rates from RAINS differ from the deposition rates from Dentener (personal communication), as used in the BAU scenario described above. For reasons of consistency, we compare N-model results for the NDEP scenario to N-model results for a slightly different BAU scenario, which is using uncontrolled $NO_y$ deposition linearly extrapolated from RAINS as N-model input, as opposed to Dentener's data. For this modified BAU scenario (using RAINS deposition rates as N-model input), the calculated DIN export

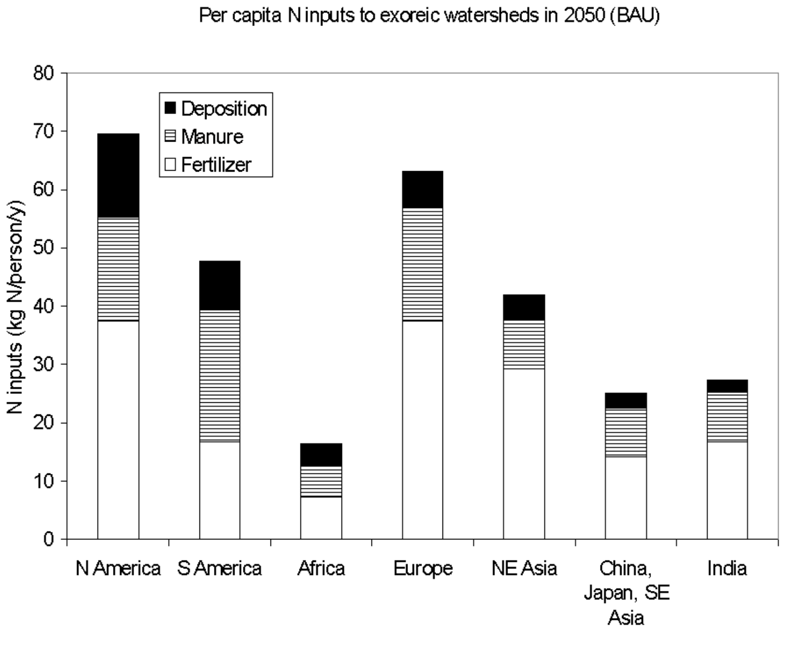

**FIGURE 1**. Per capita N inputs (fertilizer, manure, and atmospheric deposition) to exoreic watersheds by world region in 2050 for the Business-as-Usual scenario. See text for details. Definition of regions is as in Kroeze and Seitzinger[5].

rates by European rivers are in total, 4 to 6% lower than for the original BAU scenario.

Box 1. Scenario Overview

Business-as-Usual scenario (BAU): from Kroeze and Seitzinger [5]. This scenario assumes that current trends continue between 1990 and 2050. This implies that, in exoreic watersheds worldwide, the human population, fertilizer use, and atmospheric deposition of nitrogen oxides will increase by 60, 145, and 70%, respectively.

Low N diet scenario (DIET): This scenario explores the potential effect of a change in the human diet on DIN export by rivers and the associated $N_2O$ emissions. The change in diet is only assumed for industrialized regions and includes a moderate shift from animal to plant protein. This would reduce the overall N inputs to soils (in exoreic watersheds) in industrialized regions to 40 kg N/person/year by 2050.

Scenario for low $NO_y$ deposition in Europe (NDEP): The impact of $NO_x$ emission control in Europe on DIN export rates by European rivers is explored, while accounting for regional differences in emission control and resulting deposition rates. This was done by linking the regionally specific acidification model, RAINS, to the N-model[14].

## RESULTS AND DISCUSSION

### The Business-as-Usual and the DIET Scenarios

In the BAU scenario, the number of people living in watersheds that drain into oceans and large seas increases from 5 billion in 1990 to 8.5 billion in 2050, while fertilizer use and $NO_y$ deposition rates increase to 182 and 39 Tg N/year, respectively (Table 1). As a result of increased N inputs to the watersheds, the amount of DIN transport by rivers is calculated to increase from 21 Tg N/year in 1990 to 47 Tg N/year in 2050. The increase between 1990 and 2050 is particularly large for rivers draining into the North Pacific and Indian Ocean (Fig. 2).

Changes in the human diet, as assumed in the DIET scenario, are calculated to reduce total 2050 synthetic fertilizer use by 16%, relative to the BAU scenario (Table 1). The N-model calculated DIN export rates by rivers are 9% lower than the BAU level, and associated $N_2O$ emissions are 12% lower. Thus, changes in the human diet in industrialize regions have a moderate effect on worldwide N fluxes.

On a regional basis, however, the assumed changes in the human diet may reduce DIN loadings to aquatic systems considerably. Relatively large effects are calculated for the North Atlantic and European Seas (Fig. 2). Lower fertilizer use in North America and Canada is calculated to reduce DIN inputs to the western North Atlantic by 26%, relative to the BAU scenario (Table 2). The calculated $N_2O$ emissions from rivers and estuaries are 36% lower than BAU levels. Reduced fertilizer use in Europe affects DIN inputs to the eastern part of the North Atlantic Ocean (31% lower than BAU) and the European Seas (26 to 35% lower). Aquatic $N_2O$ emissions are most reduced in watersheds draining into the Baltic Sea (77%), Black Sea (67%), and the eastern North Atlantic (54%). The assumed dietary changes for Japan, Australia, and New Zealand do not have a large effect on DIN input rates to the North and South Pacific Ocean, because these countries have a relatively small share in total DIN inputs.

### The Effect of $NO_x$ Emission Control in Europe on DIN Export by Rivers

The technical potential to reduce $NO_x$ emissions in Europe is considerable. The assumed emission control in the NDEP scenario is calculated to reduce $NO_y$ deposition rates by about 80%, relative to the BAU scenario. These large reductions in deposition reduce total DIN export by European rivers by only 8%, relative to BAU levels (Table 3). Largest reductions in DIN export rates are calculated for the Neva (20%) and the Zapadnaya Dvina (13%), draining into the Baltic Sea, and for the Ebro (14%) (Table 4). For most rivers, however, the calculated effect on DIN export is 10% or less. The calculated decreases in DIN export are likely maximum estimates of decrease. This is because we used RAINS N deposition rates for 2010 that would result from maximum emission control in electricity generation, transport, and industry and compared that to a BAU scenario for 2050. The relatively small effect on river DIN export of controls on atmospheric N deposition is consistent with N inputs to European soils,

**TABLE 1**
**Worldwide Totals for N-Model Inputs of Population, Fertilizer Use, and $NO_y$ Deposition Rates in Exoreic Watersheds. Calculated DIN Export Rates by Rivers and Associated $N_2O$ Emissions in Rivers and Estuaries, for the Year 1990 and Two Scenarios For 2050 (Units: Tg N/Year Unless Mentioned Otherwise)**

| | Population (billion) | Fertilizer Use | $NO_y$ Deposition | DIN Export | $N_2O$ Emissions |
|---|---|---|---|---|---|
| 1990[a] | 4.9 | 74 | 23 | 21 | 1.3 |
| 2050 BAU[b] | 8.5 | 182 | 39 | 47 | 4.2 |
| 2050 DIET | 8.5 | 152 | 39 | 43 | 3.7 |

[a] From Seitzinger and Kroeze[3].
[b] From Kroeze and Seitzinger[5].

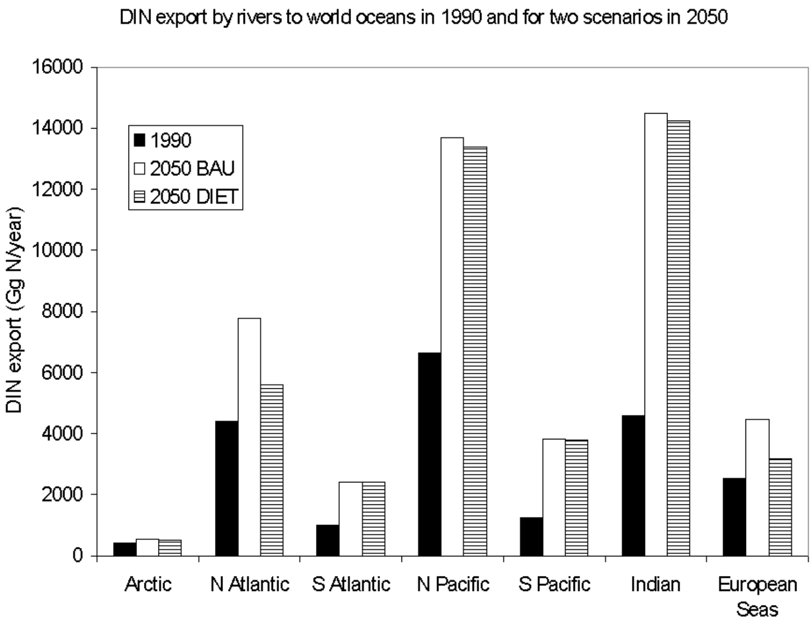

**FIGURE 2**. DIN export rates by rivers to the world's oceans in 1990 and for two scenarios in 2050. See text for scenario description. Definition of regions is as in Seitzinger and Kroeze[3].

## TABLE 2
## N-Model Inputs of Population, Fertilizer Use, and $NO_y$ Deposition Rates in Exoreic Watersheds, Summarized by Oceanic Region. Calculated DIN Export Rates by Rivers and Associated $N_2O$ Emissions in Rivers and Estuaries, for Two Scenarios for 2050

| | BAU and DIET Scenario[b] | | BAU Scenario[b] | | | | DIET Scenario | | | |
|---|---|---|---|---|---|---|---|---|---|---|
| Oceanic Region[a] | Watershed Area (1000 km$^2$) | Population (millions) | Fertilizer Use (Gg N/year) | $NO_y$ Deposition (Gg N/year) | DIN Export by Rivers (Gg N/year) | $N_2O$ Rivers + Estuaries (Gg N/year) | Fertilizer Use (Gg N/year) | $NO_y$ Deposition (Gg N/year) | Reduction in River DIN Export (% relative to BAU) | Reduction in $N_2O$ Emissions (% relative to BAU) |
| Arctic | 20730 | 70 | 2610 | 3101 | 533 | 7 | 2342 | 3101 | 4 | 4 |
| N-Atlantic West | 16612 | 557 | 20317 | 6483 | 4519 | 254 | 9421 | 6483 | 26 | 36 |
| N-Atlantic East | 7131 | 841 | 11445 | 2128 | 3262 | 280 | 6437 | 2128 | 31 | 54 |
| S-Atlantic West | 13670 | 354 | 3888 | 3621 | 1774 | 48 | 3888 | 3621 | 0 | 0 |
| S-Atlantic East | 5744 | 318 | 1628 | 2227 | 629 | 15 | 1628 | 2227 | 0 | 0 |
| N-Pacific West | 11661 | 1876 | 50516 | 5838 | 12427 | 1358 | 49941 | 5838 | 2 | 1 |
| N-Pacific East | 6001 | 185 | 4205 | 1045 | 1275 | 97 | 3554 | 1045 | 10 | 2 |
| S-Pacific West | 8924 | 456 | 6277 | 807 | 3352 | 303 | 5963 | 807 | 1 | 0 |
| S-Pacific East | 1167 | 37 | 938 | 159 | 459 | 6 | 938 | 159 | 0 | 0 |
| Indian Ocean | 17195 | 2960 | 48698 | 6892 | 14497 | 1612 | 46355 | 6892 | 2 | 2 |
| Baltic Sea | 2084 | 92 | 3568 | 1104 | 730 | 26 | 1904 | 1104 | 33 | 77 |
| Black Sea | 2524 | 191 | 9687 | 1651 | 1453 | 138 | 6238 | 1651 | 35 | 67 |
| Caspian Sea | 2881 | 102 | 4358 | 1282 | 530 | 7 | 3594 | 1282 | 12 | 12 |
| Mediterranean | 5609 | 454 | 13601 | 2198 | 1736 | 37 | 9768 | 2198 | 26 | 42 |
| Total | 121933 | 8493 | 181736 | 38536 | 47176 | 4187 | 151972 | 38536 | 9 | 10 |

[a]Definition of regions as in Seitzinger and Kroeze[3].
[b]From Kroeze and Seitzinger[5].
[c]See text for scenario description.

in that N inputs from the atmosphere have a moderate share in total N inputs, while most N inputs to soils are associated with agriculture. The effect on aquatic emissions of $N_2O$ may be somewhat larger, because of the nonlinear relationship between N inputs to aquatic systems and associated emissions of $N_2O$ as assumed in the model[3].

**TABLE 3**
**Inputs to N-Model (Watershed Area, Population, Fertilizer Use, and $NO_y$ Deposition Rates) and Calculated DIN Export Rates for European Rivers for a BAU Scenario and NDEP Scenario for 2050. Based on Domingues (2001)[14]; See Text for Scenario Description**

| Oceanic Region[a] | Watershed Area (1000 km²) | Population (millions) | Fertilizer Use (Gg N/year) | BAU $NO_y$ Deposition[d] (Gg N/year) | BAU DIN Export by Rivers (Gg N/year) | NDEP $NO_y$ Deposition[e] (Gg N/year) | NDEP DIN Export by Rivers (Gg N/year) | Reduction in NDEP (% of BAU) | |
|---|---|---|---|---|---|---|---|---|---|
| | | | | | | | | $NO_y$ | DIN |
| N-Atlantic East[b] | 1741 | 194 | 8103 | 1061 | 2207 | 163 | 2006 | 85 | 9 |
| Baltic Sea | 2084 | 92 | 3568 | 721 | 674 | 137 | 595 | 81 | 12 |
| Black Sea | 2524 | 191 | 9687 | 651 | 1349 | 183 | 1284 | 72 | 5 |
| Mediterranean[c] | 2873 | 193 | 7845 | 661 | 1155 | 141 | 1090 | 79 | 6 |
| Total | 9222 | 670 | 29202 | 3093 | 5385 | 624 | 4975 | 80 | 8 |

[a] Definition of regions based on Kroeze and Seitzinger[17].
[b] European rivers draining into N-Atlantic East, excluding Iceland, updated model results.
[c] Excluding Nile.
[d] Linearly extrapolated from no control scenario 1990–2010 from RAINS; may therefore differ from Table 2.
[e] Based on maximum feasible reduction scenario from RAINS for 2010 (see text).

**TABLE 4**
**Results for Selected Rivers in Europe. N-Model Inputs of Watershed Area, Population, Fertilizer Use, and $NO_y$ Deposition Rates. Calculated DIN Export Rates for a BAU Scenario and NDEP Scenario for 2050. Based on Domingues[14]; See Text for Scenario Description**

| | BAU and NDEP Scenario | | | BAU Scenario | | NDEP Scenario | |
|---|---|---|---|---|---|---|---|
| River[a] | Watershed Area (km²) | Population (millions) | Fertilizer Use (Gg N/year) | $NO_y$ Deposition[b] (Gg N/year) | DIN Export by Rivers (Gg N/year) | Reduction in NOy Deposition[c] (% relative to BAU) | Reduction River DIN Export (% relative to BAU) |
| Elbe | 138872 | 22 | 992 | 123 | 201 | 82 | 8 |
| Garonne | 96654 | 8 | 414 | 36 | 94 | 84 | 6 |
| Loire | 109297 | 9 | 757 | 50 | 125 | 83 | 5 |
| Rhine | 200949 | 37 | 1068 | 215 | 392 | 84 | 12 |
| Neva | 268390 | 8 | 174 | 80 | 42 | 80 | 20 |
| Oder | 121859 | 17 | 398 | 83 | 51 | 81 | 10 |
| Vistula | 213335 | 24 | 684 | 125 | 90 | 80 | 10 |
| Zapadnaya Dvina | 117209 | 4 | 209 | 45 | 29 | 78 | 13 |
| Danube | 757896 | 76 | 3562 | 371 | 752 | 80 | 7 |
| Dnepr | 525095 | 39 | 1826 | 120 | 130 | 68 | 3 |
| Dnestr | 107073 | 9 | 333 | 28 | 44 | 73 | 5 |
| Don | 428072 | 13 | 1585 | 57 | 67 | 56 | 2 |
| Ebro | 101354 | 5 | 160 | 33 | 36 | 85 | 14 |
| Rhone | 103301 | 11 | 488 | 69 | 148 | 84 | 10 |

[a] Rivers as in Kroeze and Seitzinger[17].
[b] Linearly extrapolated from no-control scenario 1990 to 2010 from RAINS.
[c] Based on maximum feasible reduction scenario from RAINS for 2010 (see text).

Although the calculated effects of $NO_x$ emission control on riverine DIN (8%) may seem small compared to the effect on $NO_y$ deposition (about 80%), they may contribute to European policies on coastal eutrophication. For instance, European countries aim at reducing N inputs to the maritime area by 50%, relative to 1985, as agreed under the OSPAR Convention for the Protection of the Maritime Environment of the North-East Atlantic[15,16]. The BAU scenario shows that, without policies to reduce N inputs to aquatic systems, DIN export by rivers to the North Sea (roughly the N-Atlantic East oceanic region in Table 3), may increase from about 1.8 Tg N/year in 1990 to 2.2 Tg N/year in 2050. Current policy plans to reduce the N inputs to the North Sea are ignored in the BAU scenario, and focus mainly on point sources and agricultural leaching losses of N. The comparisons of the BAU and DIET scenarios indicate that a reduction in fertilizer use, such as that associated with a change in human diet based on less protein, indeed could reduce N inputs to the North Sea considerably. Our analysis also shows that $NO_x$ emission control may potentially avoid 9% of the 2050 DIN inputs to the North Sea. Thus, policies primarily aimed at air pollution control may, as a side effect, help achieving the OSPAR convention targets.

## Uncertainties

This is the first attempt, as far as we are aware, to estimate spatially explicit global changes in N export by world rivers and associated $N_2O$ emissions due to changes in the human diet or changes in $NO_y$ deposition associated with emission controls on fuel combustion. Admittedly, there are many uncertainties in the model projections for these scenarios. These uncertainties are associated both with the assumptions made in the development of the scenarios, as well as with the input data used to run the scenarios, as noted throughout the text. In addition, there are uncertainties associated with the N model itself, as discussed in previous publications[3,5]. Despite these uncertainties, the model results provide a first insight into the potential positive effects that changes in human behavior could exert on N export to coastal ecosystems and associated $N_2O$ emissions. Future analyses will undoubtedly improve upon this initial work as advances are made in understanding land-atmosphere-ocean interactions of N.

## ACKNOWLEDGEMENTS

This study was in part funded through the Netherlands Organization for Scientific Research (NWO) (CK) and NOAA New Jersey Sea Grant and NJDEP (SPS; NJSG-01-469). We gratefully acknowledge Nina Caraco, John Cole, Lex Bouwman, Frank Dentener, J. Graham Cogley, and others, who provided information or assistance in developing the original model[3]. We also acknowledge Lex Bouwman, Oene Oenema, and an anonymous reviewer for their comments on an earlier version of this manuscript.

| | | |
|---|---|---|
| Received: | July | 27, 2001 |
| Revised: | October | 3, 2001 |
| Accepted: | October | 5, 2001 |
| Published: | November | 2, 2001 |

## BIOSKETCHES

**Carolien Kroeze** is at the Environmental Systems Analysis Group of the Department of Environmental Sciences, Wageningen University, The Netherlands. Her field of study includes biogeochemistry and environmental systems analysis. She has been involved in integrated assessment studies, with special emphasis on the modelling of emissions of air pollutants for scenario analyses and evaluation of environmental policy, including the effects of acid rain abatement on greenhouse gas emissions.

**Sybil Seitzinger** is Director of the Rutgers/NOAA Cooperative Marine Education and Research Program at Rutgers University, and is on the faculty in the Institute of Marine and Coastal Sciences. Her research addresses sources, effects, and fate of nitrogen in aquatic ecosystems. Her current research activities include: 1) dissolved organic nitrogen chemical characterization, inputs, and bioavailability in aquatic ecosystems; 2) denitrification in rivers, estuaries, and continental shelves; and 3) global modeling of N transport by world rivers to coastal ecosystems.

**Rita Domingues** graduated at Aveiro University (Portugal) in Environmental Engineering and holds an M. Sc. degree in Environmental Science from Wageningen University (The Netherlands). The subject for her graduation report dealt was a Life Cycle Assessment of a 3- × 300-MW Combined Cycle Power Plant. Her M.Sc. thesis was entitled: Future trends in nitrogen transport in terrestrial and aquatic systems in Europe: Linking RAINS to the N-model. Presently she works on the environmental certification project (ISO 14001) for the Carregado Power Plant, in Portugal.
